# Development of Corneal Astigmatism (CA) according to Axial Length/Corneal Radius (AL/CR) Ratio in a One-Year Follow-Up of Children in Beijing, China

**DOI:** 10.1155/2018/4209236

**Published:** 2018-08-30

**Authors:** Fenglei Wang, Lin Xiao, Xuxia Meng, Ling Wang, Dabo Wang

**Affiliations:** ^1^Department of Ophthalmology, The Affiliated Hospital of Qingdao University, Qingdao, China; ^2^Department of Ophthalmology, Beijing Shijitan Hospital, The Ninth Clinical Medical College of Beijing University, Beijing, China

## Abstract

**Purpose:**

The correlations between the axial length-to-corneal radius (AL/CR) ratio and corneal astigmatism (CA) were studied by prospectively analyzing and comparing survey data from school children in the Beijing urban area from 2014 to 2015.

**Methods:**

In this longitudinal study, a total of 2,970 students were enrolled in 2014, and 2,179 students were enrolled in 2015. The students were in grades 1 and 4 of primary schools located in the Yangfangdian district of Beijing. The students were examined using the standard logarithmic visual acuity chart for uncorrected visual acuity (UCVA) and IOLMaster for ocular components.

**Results:**

From 2014 to 2015, the students from grades 1 and 4 had significantly worse UCVA results, longer axial lengths (AL), and greater AL/CRs (*p* < 0.001). The boys had a longer AL and corneal radius (CR) than the girls (*p* < 0.001). A significantly higher rate of increased CA was observed for the students with increased AL/CR than for those with decreased or unchanged ratios (AL/CR for grade 1, *X*^2^ = 12.304, *p*=0.001; for grade 4, *X*^2^ = 29.044, *p* < 0.001). In addition, with increased AL/CR over one year, the CA value of the students in grades 1 and 4 became significantly larger (grade 1, *p*=0.001; grade 4, *p* < 0.001); moreover, the UCVA became worse (*p* < 0.001).

**Conclusions:**

We found that UCVA and AL growth were affected by aging. An increase in the AL/CR ratio is a risk factor for the progression of corneal astigmatism for school children.

## 1. Introduction

Astigmatism is a common refractive anomaly that is described clinically as a bivariate quantity consisting of an astigmatic modulus and an axis. Children with uncorrected high astigmatism have an increased risk of developing refractive amblyopia [1]. According to several studies, the optical blur imposed by astigmatism may also predispose patients to myopia development [2, 3]. Thus, astigmatism is an important clinical and public health problem.

Two components of astigmatism can be independently measured: refractive (total) astigmatism (RA) and corneal astigmatism (CA). The relationship between RA and CA has been previously described as Javal's rule [4–6]. Longitudinal studies have shown that the early cylindrical error is greatly reduced or eliminated during the first two years of life [7–9], followed by slower changes occurring between ages 2 and 6 years [10] and stability between the ages of 6 and 12 years [11]. The positive compensatory changes in corneal and internal astigmatisms accompany a fast reduction in refractive astigmatism in infancy and early childhood [12]. Numerous reports have linked astigmatism to the development and progression of myopia in children [13]. Changes in astigmatism must be associated with the development of ocular biometry and structure. Thus, the determination of a target for estimating the tendency of astigmatism is quite critical in the prediction of myopic progression.

Many authors have found that the most myopic subjects, especially those with greater ALs, have smaller corneal radii [13–16]. Grosvenor et al. [5, 17] explored the link between myopia and the ratio between the AL and CR (AL/CR) of the eye. A high AL/CR ratio in an emmetropic person indicates that a reduction in the power of the crystalline lens may compensate for the increased AL. Goss and Jackson [18] found that a high AL/CR ratio in an emmetrope was a risk factor for developing myopia because the crystalline lens was close to the limit of its emmetropizing capacity and would have difficulty flattening any further. Therefore, in emmetropic subjects, a relationship between the AL/CR ratio and the thickness of the crystalline lens likely exists. However, a relationship between the AL/CR ratio and CA has not been definitively demonstrated.

In this study, we sought to (1) report and compare the distribution of corneal astigmatism, axial length, and AL/CR ratio in grade 1 and 4 school children in 2014 and 2015, (2) compare the differences of ocular biometric parameters in students of different genders, and (3) document the effectiveness of the AL/CR ratio in anticipation of corneal astigmatism development.

## 2. Materials and Methods

### 2.1. Study Population

The Beijing Yangfangdian refraction study is a survey of refractive errors and other eye diseases in a large sample of grade 1 (5–8 years old) and grade 4 (9–11 years old) school children from 7 elementary schools of urban center districts in Beijing. A 1-year follow-up study will be conducted to reexamine these children when they are in grades 2 and 5. And they are the same evaluated both times in a repeated manner. A total of 2970 students in 2014 and 2179 students in 2015 were selected and examined (Table 1). In 2014, 1282 students from grade 1 (658 boys and 624 girls, average age 6.44 ± 0.52 years) and 1688 students from grade 4 (892 boys and 796 girls, average age 9.25 ± 0.46 years) participated in the study. In 2015, 1173 students from grade 2 (596 boys and 577 girls, average age 7.44 ± 0.51 years) and 952 students from grade 5 (496 boys and 456 girls, average age 10.25 ± 0.48 years) participated in the study. Only the data collected from 2125 students who were examined both years underwent paired-sample statistical analysis.

### 2.2. Examination and Calculations

The study was approved by the Ethics Committee of the Affiliated Hospital of Qingdao University and the Beijing Shijitan Hospital and was conducted in accordance with the principles of the Declaration of Helsinki. Informed written consent was obtained from at least one parent of each participating child. Verbal consent was also obtained from the children before the examination occurred.

Monocular UCVA was tested by an ophthalmic technologist using a standard logarithmic visual acuity chart (Yuehua Medical Apparatus and Instruments, Inc., Shantou, Guangdong, China) at 5 m. Luminance values were within the recommendations (160 cd/m^2^) for standardizing the UCVA measurement [19]. All the subjects were asked to identify each letter individually, starting with the upper left-hand letter and reading along the line and then proceeding to the next line until they could no longer correctly name at least one letter on the line. The students were instructed to read slowly and guess the letters when they were unsure. The rule for stopping was four or five mistakes on a line [20]. Children with glasses were asked to remove the glasses for the visual measurements. The right eye was tested first, followed by the left eye, and the final visual acuity results were reported in decimals.

AL and keratometry (*K*) measurements [21] were performed using the IOLMaster (Carl Zeiss Meditec AG, Jena, Germany). Each keratometry reading was the average of five internal measurements made by the instrument. Three consistent readings were obtained in which CA did not vary by >0.10 diopters (D) between readings, and the astigmatic axis varied by ≤5° for astigmatism ≥0.50 D and by ≤10° for astigmatism >0.50 D. The mean value of these readings was used for the analysis. Samples of astigmatism type changes over tow years were excluded. CR was calculated using the keratometer index of 1.3375. AL was measured 5 times, and the median value of the five readings was used for analysis.

CA was calculated as *K*_min_ − *K*_max_, where *K*_min_ represents the meridian with the least refractive power and *K*_max_ represents the meridian with the greatest refractive power [21]. The corneal cylinder axis was set along the *K*_min_ median. The axial length-to-corneal radius (AL/CR) ratio was defined as the axial length divided by the mean corneal radius.

Data from the right and left eyes were analyzed separately, but only the results of the right eyes are presented because no difference between the results for the right and left eyes was observed.

### 2.3. Data Analysis

Statistical analyses were performed using a software package (SPSS 20.0). A paired-samples *t*-test was used for comparison of the 2-year data; however, an independent-samples *t*-test was used for the gender comparisons. Chi-square statistics were used to test whether the increases in CA were the same for the grade 1 and 4 students who had increased or decreased AL/CR over the two-year study period. Moreover, the RR and OR values were calculated and exported. The *p* values are reported as two-sided and were considered statistically significant when the values were less than 0.05.

## 3. Results

Table 2 displays the visual acuity and ocular biometric data comparison of the grade 1 and 4 samples collected in 2014 and 2015. Regarding the students in grades 1 and 4, significant differences were observed between the data from 2014 to 2015 in terms of the UCVA (*p* < 0.001), AL (*p* < 0.001), and AL/CR (*p* < 0.001). However, no significant differences were observed regarding the *K*, CA, and CR values (*p* > 0.05). Between 2014 and 2015, the UCVA decreased, AL increased, and the AL/CR ratio increased significantly; however, CA and *K* did not show any significant changes.

Table 3 shows the oculometric parameters for the boys and girls in grade 1 in 2014 and 2015. Significant differences were observed in the CA (2014, *p*=0.036; 2015, *p*=0.008), AL (*p* < 0.001), *K* (*p* < 0.001), CR (*p* < 0.001), and AL/CR (2014, *p* < 0.001; 2015, *p* < 0.001) between 2014 and 2015. The boys had obviously smaller CA and *K*, longer AL and CR, and larger AL/CR values than the girls in 2014 and 2015. However, no significant difference was found in the UCVA (*p* > 0.05).

Table 4 shows the oculometric parameters for the boys and girls in grade 4 in 2014 and 2015. Significant differences in the UCVA (2014, *p* < 0.001; 2015, *p*=0.002), AL (*p* < 0.001), *K* (*p* < 0.001), and CR (*p* < 0.001) values were found between 2014 and 2015. The boys had a better UCVA, smaller *K*, and longer AL and CR than the girls in 2014 and 2015. However, no significant differences were observed in CA (*p* > 0.05). For the AL/CR values (2014, *p*=0.040; 2015, *p*=0.101), the boys showed partial differences compared with the girls in 2014 and 2015.

Tables 5 and 6 show the changes in CA in the grade 1 and 4 students between 2014 and 2015. In this study, we set increased AL/CR between 2014 and 2015 as the exposure factor resulting in the increased CA value (positive CA) and then set decreased or unchanged AL/CR between 2014 and 2015 as the nonexposed factor resulting in a decreased or unchanged CA value (negative CA). Based on the calculation of the odds ratio (OR) and relative ratio (RR), we estimated the associated magnitude between AL/CR and CA.

Tables 5 and 6 indicate that among the students who had increased CA values, significantly more showed increased AL/CR than decreased or unchanged AL/CR (grade 1, *X*^2^ = 12.304, *p*=0.001; grade 4, *X*^2^ = 29.044, *p* < 0.001). Moreover, OR = 1.861 and RR = 1.405 for the grade 1 students and OR = 2.370 and RR = 1.530 for the grade 4 students, demonstrating a moderate association between the exposure factor (increased AL/CR) and a positive result (increased CA).

Table 7 displays the UCVA and CA comparisons for the grade 1 and 4 students whose AL/CR changed between 2014 and 2015. The results indicated that when the AL/CR increased from 2014 to 2015, the UCVA became significantly worse (*p* < 0.001), and the CA became significantly larger (grade 1, *p* < 0.001; grade 4, *p* < 0.001). However, when the AL/CR decreased or did not change, the CA became significantly smaller (grade 1, *p*=0.027; grade 4, *p* < 0.001); additionally, the UCVA became significantly worse (*p* < 0.001) for the grade 4 students, but for the grade 1 students, no significant difference in UCVA was observed (*p*=0.977).

**Figure 1:**
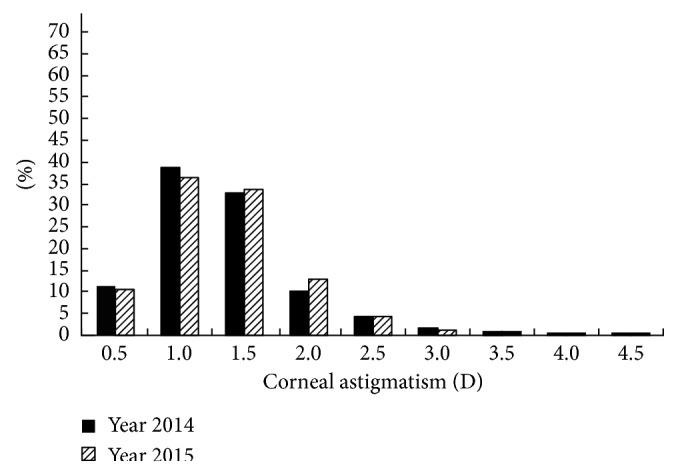
Distributions of the prevalence of CA for grade 1 students in 2014 and 2015.

**Figure 2:**
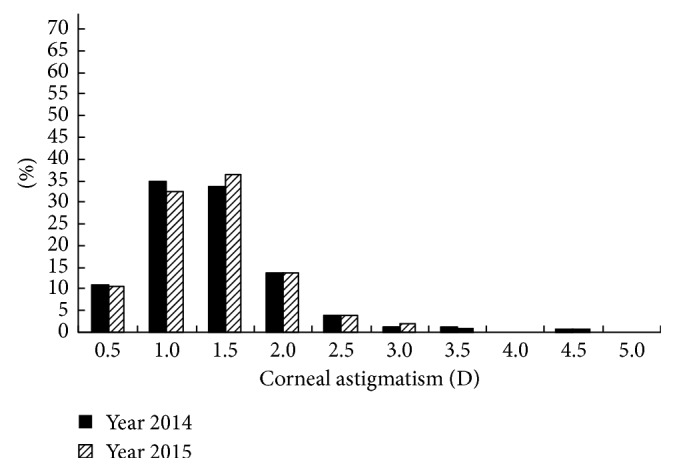
Distributions of the prevalence of CA for grade 4 students in 2014 and 2015.

**Figure 3:**
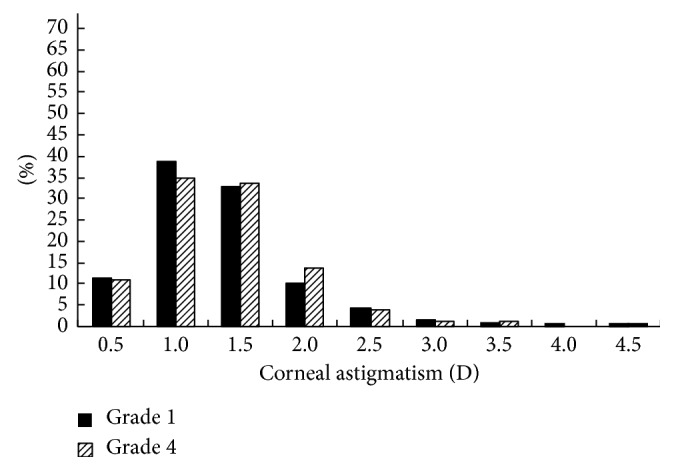
Distributions of the CA prevalence in grade 1 and 4 students in 2014.

**Figure 4:**
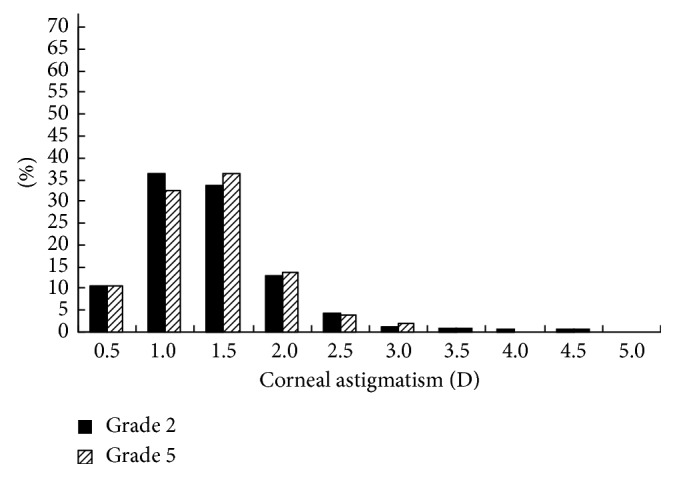
Distributions of the CA prevalence in grade 2 and 5 students in 2015.

The distributions of the prevalence of CA for the students in grades 1 and 4 in 2014 and 2015 are shown in Figures 1–4. Statistically, the students in both grades had statistically very similar values for 2014 and 2015. The CA distributions were mainly centralized in the range from 0.5 to 1.5 D.  Figure 1 shows that the percentage of grade 1 students with CA < 1.0 D decreased from 2014 to 2015, while the percentage of students with CA = 1.0–2.0 D increased, indicating that the CA of several students had changed slightly in one year. Moreover, Figure 2 shows the same situation for grade 4 students.  Figure 3 shows that, in 2014, the percentage of grade 2 students with CA < 1.0 D was larger than the percentage of grade 4 students; however, for CA = 1.0–2.0 D, the percentage decreased.  Figure 4 shows the same situation for 2015.

## 4. Discussion

The oculometric parameters observed in our study, including AL, CR, *K*, and AL/CR values, were in general agreement with the values determined by others for population samples of a similar age [22–27]. We found that UCVA, AL, and AL/CR grew significantly with age; however, the CR, *K*, and CA values were not significantly different between the two evaluation timepoints of our study (Table 2).

Our data for grade 4 also confirmed significant differences in AL and CR according to the gender. Consistent with previous reports, AL for boys was found to be approximately 0.5 mm greater than the AL for girls [22, 24], and the cornea was flatter for boys than for girls. Furthermore, the boys in grade 4 presented higher UCVA than girls (Table 4).

Juvenile-onset myopia usually begins between the ages of 6 and 14 years and stops or slows as soon as the general physical growth stage finishes at the end of adolescence [28–30]. This type of myopia is thought to occur because the elongation of the eye is insufficiently compensated by the flattening of the cornea and crystalline lens [31, 32]. The cornea appears to play an emmetropizing role in preserving emmetropia or low myopia. This emmetropizing capacity may be insufficient when there is excessive axial lengthening of the ocular globe, resulting in the appearance of myopia [33]. Yebra-Pimentel et al. [34] reported that an AL/CR ratio above 3 in an emmetropic or mildly myopic eye would indicate that the cornea has reached its limit of compensatory capacity in the face of further AL increases. Although previous researchers have confirmed that a higher AL/CR ratio was a risk factor in the development of myopia and could reflect less compensatory capacity, whether a higher AL/CR ratio is an indicator of a larger corneal astigmatism remains unclear in this study.

Our longitudinal study (Tables 5 and 6) showed a significantly higher occurrence of increased CA for the students with a increased AL/CR than for those with a decreased or unchanged AL/CR (grade 1, *X*^2^ = 12.304, *p*=0.001; grade 4, *X*^2^ = 29.044, *p* < 0.001). The OR and RR values showed a moderate association between the exposure factor (increased AL/CR) and a positive result (increased CA). Thus, we demonstrated that an increased AL/CR ratio was a risk factor for CA progression.

For both grade 1 and 4 students who exhibited increased AL/CR, the VA decreased, and CA increased significantly (*p* < 0.001; Table 7). The larger CA may indicate the extent to which an imbalance between axial elongation and corneal curvature contribute to astigmatism progression. The declining UCVA may be the result of the mutual development of astigmatism and myopia.

Our study aimed at identifying the relationship between AL/CR and CA as the students' ages increased to monitor the developmental tendency of CA and UCVA. We hypothesized that the increases in AL/CR may result from the lengthening of the AL and the shortening of the CR, both of which could increase the pressure of the eyelids on the eye globes and ultimately lead to a corneal deformation. Accordingly, the CA value became larger as the students aged.

The cross-sectional and longitudinal comparisons of different CA prevalence values (Figures 1–4) indicated that the CA prevalence distributions were generally similar. This result further indicates that, for the total population, the CA value remained relatively stable, irrespective of aging.

This study has several limitations. A linear relationship exists between corneal astigmatism and refractive astigmatism [4]; therefore, we only chose CA as a representative target for the statistical analysis. In addition, only 7 schools from one district in the Beijing urban area were chosen, and the results from the district may not be sufficiently representative of the entire city. Thus, in future studies, a larger population with more extensive representation of the Beijing urban area will be investigated and followed up for a longer period. In summary, we found that UCVA and AL growth were affected by age, and an increase in the AL/CR ratio is a risk factor for the progression of corneal astigmatism of school children.

## Figures and Tables

**Table 1:** The distributions of students in the two surveys.

Age (years)	Year 2014		Year 2015	
	*N*	(%)	*N*	(%)
5	11	0.37	0	0
6	705	23.74	5	0.23
7	561	18.89	676	31.02
8	18	0.61	523	24.00
9	1243	41.85	19	0.87
10	425	14.31	709	32.54
11	7	0.23	241	11.06
12	0	0	6	0.28
Total	2970		2179	

**Table 2:** Oculometric parameters of the students in grades 1 and 4 in 2014 and 2015 ($\bar{x}\pm s$) (*n*).

	Grade 1 (aged 5–8 years)	Grade 4 (aged 9–11 years)
UCVA ± *s* (*n*)		
Year 2014	1.02 ± 0.26 (1167)	0.83 ± 0.43 (769)
Year 2015	0.92 ± 0.30 (1167)	0.64 ± 0.37 (769)
*p*	0.001^*∗*^	0.001^*∗*^
95% CI	−0.12∼−0.08	−0.21∼−0.16
AL ± *s*mm (*n*)		
Year 2014	22.73 ± 0.73 (1161)	23.65 ± 0.93 (942)
Year 2015	23.14 ± 0.86 (1161)	24.03 ± 1.02 (942)
*p*	0.001^*∗*^	0.001^*∗*^
95% CI	0.38∼0.44	0.35∼0.41
*K* ± *s*D (*n*)		
Year 2014	43.29 ± 1.46 (1078)	43.29 ± 1.44 (850)
Year 2015	43.30 ± 1.45 (1078)	43.31 ± 1.43 (850)
*p*	0.768	0.710
95% CI	−0.14∼0.10	−0.09∼0.13
CA ± *s*D (*n*)		
Year 2014	1.11 ± 0.62 (1078)	1.13 ± 0.59 (850)
Year 2015	1.13 ± 0.59 (1078)	1.15 ± 0.59 (850)
*p*	0.440	0.311
95% CI	−0.03∼0.07	−0.02∼0.07
CR ± *s*mm (*n*)		
Year 2014	7.80 ± 0.26 (1078)	7.80 ± 0.26 (850)
Year 2015	7.81 ± 0.26 (1078)	7.80 ± 0.26 (850)
*p*	0.791	0.716
95% CI	−0.02∼0.02	−0.02∼0.02
AL/CR ± *s* (*n*)		
Year 2014	2.91 ± 0.17 (1078)	3.03 ± 0.12 (849)
Year 2015	2.97 ± 0.11 (1078)	3.08 ± 0.15 (849)
*p*	0.001^*∗*^	0.001^*∗*^
95% CI	0.05∼0.07	0.04∼0.06

*s* = standard deviation; mm = millimeter; D = diopter; ^*∗*^*p* < 0.001.

**Table 3:** Oculometric parameters for male and female school children in grade 1 (aged 5–8 years) in 2014 and 2015 ($\bar{x}\pm s$) (*n*).

Grade 1	Boys	Girls	*p*	95% CI
UCVA ± *s* (*n*)				
Year 2014	1.02 ± 0.28 (651)	1.01 ± 0.25 (630)	0.350	−0.01∼0.04
Year 2015	0.92 ± 0.30 (592)	0.93 ± 0.29 (575)	0.484	−0.02∼0.04
AL ± *s*mm (*n*)				
Year 2014	23.00 ± 0.67 (647)	22.44 ± 0.66 (623)	0.001^*∗*^	0.49∼0.63
Year 2015	23.40 ± 0.81 (592)	22.87 ± 0.83 (577)	0.001^*∗*^	0.43∼0.62
*K* ± *s*D (*n*)				
Year 2014	43.26 ± 1.46 (632)	43.38 ± 1.43 (620)	0.001^*∗*^	−0.28∼0.05
Year 2015	42.92 ± 1.40 (556)	43.65 ± 1.41 (547)	0.001^*∗*^	−0.89∼−0.56
CA ± *s*D (*n*)				
Year 2014	1.07 ± 0.59 (632)	1.14 ± 0.63 (632)	0.036	−0.15∼−0.01
Year 2015	1.08 ± 0.57 (556)	1.18 ± 0.61 (547)	0.008	−0.17∼−0.03
CR ± *s*mm (*n*)				
Year 2014	7.86 ± 0.26 (632)	7.74 ± 0.24 (620)	0.001^*∗*^	0.10∼0.15
Year 2015	7.87 ± 0.26 (556)	7.74 ± 0.25 (547)	0.001^*∗*^	0.10∼0.16
AL/CR ± *s* (*n*)				
Year 2014	2.92 ± 0.14 (632)	2.89 ± 0.21 (620)	0.001^*∗*^	0.01∼0.05
Year 2015	2.98 ± 0.11 (556)	2.96 ± 0.11 (547)	0.010	0.00∼0.03

*s* means standard deviation; ^*∗*^*p* < 0.001.

**Table 4:** Oculometric parameters for male and female schoolchildren in grade 4 (aged 9–11 years) in 2014 and 2015 ($\bar{x}\pm s$) (*n*).

Grade 4	Boys	Girls	*p*	95% CI
UCVA ± *s* (*n*)				
Year 2014	0.89 ± 0.42 (795)	0.82 ± 0.41 (699)	0.001	0.02∼0.11
Year 2015	0.71 ± 0.40 (493)	0.63 ± 0.37 (455)	0.002	0.03∼0.12
AL ± *s*mm (*n*)				
Year 2014	23.88 ± 0.88 (886)	23.41 ± 0.93 (786)	0.001^*∗*^	0.38∼0.56
Year 2015	24.24 ± 1.01 (495)	23.80 ± 1.00 (456)	0.001^*∗*^	0.31∼0.56
*K* ± *s*D (*n)*				
Year 2014	42.95 ± 1.38 (876)	43.67 ± 1.38 (783)	0.001^*∗*^	−0.90∼−0.54
Year 2015	43.05 ± 1.43 (447)	43.61 ± 1.38 (418)	0.001^*∗*^	−0.74∼−0.37
CA ± *s*D (*n*)				
Year 2014	1.11 ± 0.63 (876)	1.15 ± 0.53 (783)	0.231	−0.12∼0.03
Year 2015	1.12 ± 0.61 (447)	1.19 ± 0.56 (418)	0.107	−0.14∼0.01
CR ± *s*mm (*n*)				
Year 2014	7.87 ± 0.25 (876)	7.75 ± 0.24 (783)	0.001^*∗*^	0.09∼0.14
Year 2015	7.85 ± 0.26 (447)	7.75 ± 0.24 (418)	0.001^*∗*^	0.07∼0.13

*s* means standard deviation; ^*∗*^*p* < 0.001.

**Table 5:** The CA changes in grade 1 students from 2014 to 2015.

Year 2014–2015			
Grade 1 (aged 5–8 years)	Positive CA^*∗*^ (*n*)	Negative CA^*∗∗*^ (*n*)	Total (*n*)
AL/CR increased	488	434	922
AL/CR decreased or unchanged	58	96	154

Chi-square test: *X*^2^ = 12.304, *p*=0.001; OR = 1.861 (95% CI 1.31∼2.64); RR = 1.405 (95% CI 1.14∼1.74); ^*∗*^the number of students with increased CA; ^*∗∗*^the number of students with decreased or unchanged CA; OR means odds ratio; RR means relative ratio.

**Table 6:** The CA changes in grade 4 students from 2014 to 2015.

Year 2014–2015			
Grade 4 (ages 9–11 years)	Positive CA^*∗*^ (*n*)	Negative CA^*∗∗*^ (*n*)	Total (*n*)
AL/CR increased	387	244	631
AL/CR decreased or unchanged	85	133	218

Chi-square test: *X*^2^ = 29.044, *p* < 0.001; OR = 2.370 (95% CI 1.72∼3.26); RR = 1.530 (95% CI 1.28∼1.82); ^*∗*^the number of students with increased CA; ^*∗∗*^the number of students with decreased or unchanged CA; OR means odds ratio; RR means relative ratio.

**Table 7:** Comparisons of VA and CA in grade 1 and 4 students with AL/CR changes between 2014 and 2015 ($\bar{x}\pm s$) (*n*).

	Grade 1 (aged 5–8 years)		Grade 4 (aged 9–11 years)	
	AL/CR increased	AL/CR decreased or unchanged	AL/CR increased	AL/CR decreased or unchanged
UCVA ± *s* (*n*)				
Year 2014	1.02 ± 0.26 (918)	0.99 ± 0.26 (153)	0.84 ± 0.44 (487)	0.81 ± 0.41 (204)
Year 2015	0.91 ± 0.30 (918)	0.99 ± 0.27 (153)	0.65 ± 0.38 (487)	0.62 ± 0.36 (204)
*p*	0.001^*∗*^	0.977	0.001^*∗*^	0.001^*∗*^
95% CI	−0.13∼−0.09	−0.05∼0.05	−0.21∼−0.15	−0.23∼−0.14
CA ± *s*D (*n*)				
Year 2014	1.08 ± 0.59 (922)	1.21 ± 0.67 (154)	1.07 ± 0.54 (631)	1.31 ± 0.69 (218)
Year 2015	1.14 ± 0.59 (922)	1.06 ± 0.62 (154)	1.17 ± 0.58 (631)	1.10 ± 0.59 (218)
*p*	0.001	0.027	0.001^*∗*^	0.001^*∗*^
95% CI	0.02∼0.09	−0.27∼−0.02	0.06∼0.15	−0.33∼0.10

*s* means standard deviation; ^*∗*^*p* < 0.001.

## Data Availability

The data used to support the findings of this study are available from the corresponding author upon request.
